# Action of essential oils from Brazilian native and exotic medicinal species on oral biofilms

**DOI:** 10.1186/1472-6882-14-451

**Published:** 2014-11-18

**Authors:** Salete MF Bersan, Livia CC Galvão, Vivian FF Goes, Adilson Sartoratto, Glyn M Figueira, Vera LG Rehder, Severino M Alencar, Renata MT Duarte, Pedro L Rosalen, Marta CT Duarte

**Affiliations:** Department of Physiological Sciences, Piracicaba Dental School, University of Campinas, (UNICAMP), 901 Limeira Av, Zip Code 13414-018 Piracicaba, SP Brazil; Chemical, Biological and Agricultural Pluridisciplinary Research Center (CPQBA), University of Campinas (UNICAMP), Box. 6171, Zip Code: 13081-970 Campinas, SP Brazil; Department of Agri-food industry, Food and Nutrition, Escola Superior de Agricultura “Luiz de Queiroz”, University of São Paulo (USP), 11 Pádua Dias Avenue, Zip Code: 13418-900 Piracicaba, SP Brazil

**Keywords:** Antimicrobial activity, Essential oil and oral biofilm

## Abstract

**Background:**

Essential oils (EO) obtained from twenty medicinal and aromatic plants were evaluated for their antimicrobial activity against the oral pathogens *Candida albicans, Fusobacterium nucleatum, Porphyromonas gingivalis, Streptococcus sanguis* and *Streptococcus mitis*.

**Methods:**

The antimicrobial activity of the EO was evaluates by microdilution method determining Minimal Inhibitory Concentration. Chemical analysis of the oils compounds was performed by Gas chromatography–mass spectrometry **(**CG-MS). The most active EO were also investigated as to their actions on the biolfilm formation.

**Results:**

The most of the essential oils (EO) presented moderate to strong antimicrobial activity against the oral pathogens (MIC - Minimal Inhibitory Concentrations values between 0.007 and 1.00 mg/mL). The essential oil from *Coriandrum sativum* inhibited all oral species with MIC values from 0.007 to 0.250 mg/mL, and MBC/MFC (Minimal Bactericidal/Fungicidal Concentrations) from 0.015 to 0.500 mg/mL. On the other hand the essential oil *of C. articulatus* inhibited 63.96% of *S. sanguis* biofilm formation. Through Scanning Eletronic Microscopy (SEM) images no changes were observed in cell morphology, despite a decrease in biofilm formation and changes on biofilm structure. Chemical analysis by Gas Chromatography – Mass Spectrometry (GC-MS) of the *C. sativum* essential oil revealed major compounds derivatives from alcohols and aldehydes, while *Cyperus articulatus* and *Aloysia gratissima* (EOs) presented mono and sesquiterpenes.

**Conclusions:**

In conclusion, the crude oil from *C. articulatus* exhibited the best results of antimicrobial activity e ability to control biofilm formation. The chemical analysis showed the presence of terpenes and monoterpenes such as a-pinene, a-bulnesene and copaene. The reduction of biofilms formation was confirmed from SEM images. The results of this research shows a great potential from the plants studied as new antimicrobial sources.

## Background

The oral cavity is the habitat of several kinds of microorganisms, which form a complex community structure that can adhere to the teeth surface or to mucosal epithelial forming biofilms [1]. Microbial biofilms are communities formed when single cell microorganisms become firmly adhered to a solid surface covered by an extracellular polysaccharide matrix, and can be formed from multiple or single microbial species [2].

Oral biofilm acquires new microbial species in each stage of its development, including *Lactobacillus casei, Streptococcus sanguis, S. mutans, S. mitis* and *S. sobrinus*, which due their pathogenicity could damage the enamel and gum tissue [3]. Diseases appear in this micro-environment when there is a lack of equilibrium in the ecosystem of the bacterial biofilm formed [4], and thus the mechanical removal of the biofilm is an important factor to prevention of caries and periodontal diseases. Since biofilm is an organized association, able to adhere to teeth and causing pathological alterations in oral cavity, its disaggregation is indicated as soon as possible [5]. Considering the importance of this dissociation, it is important to associate both chemical and mechanical procedures in order to control its formation [6].

Antimicrobial substances such as chlorhexidine digluconate has been considered as golden standard when compared to other chemical agents used in dentistry, due its capacity to avoid dental biofilm formation [7]. The main advantage of using chlorhexidine is its wide antimicrobial spectrum, acting on both Gram-positive and Gram-negative microorganisms, and its prolonged and continuous effect even in the presence of blood and other body fluids [8]. However, the prolonged use of chlorhexidine can cause mucous peeling, stains on the teeth, alterations in the sense of taste, compromising of the wounds healing and reduction of fibroblast adhesion to radicular surfaces [9]. Thus a potential antimicrobial adjuvant alternative with less side-effects would be of great value acting on oral affections.

An increasing interest in natural products as a source of new bioactive molecules has been observed in the literature [10]. These include essential oils (EOs) from medicinal and aromatic plants, products of their secondary metabolism. They are characterized as having a very diverse composition derived mainly from two different groups of compounds, the terpenoids (monoterpenes and sesquiterpenes) and phenylpropanoids [11]. These compounds come from different precursors of primary metabolism and are synthesized through different pathways conferring antimicrobial and antifungal properties [12]. The antimicrobial properties of EOs against a wide variety of bacteria and fungi have been shown, including oral pathogens [10, 13–15]. Thus, the use of plants as alternative medicine has gained the attention of the scientific community, since this is a promising field for the treatment of pathogens, including those related to the oral cavity. Considering the increased interest in the use of natural products as alternative antimicrobial substances, the aim of this work was to evaluate the activity of EOs from twenty medicinal plants against both planktonic cells and biofilms of oral pathogens as well the chemical composition from the most active oils by GC/MS – Gas Chromatography/Mass Spectrometry analysis and, the possible morphological cells alterations by SEM – Scanning Electronic Microscopy.

## Methods

### Microorganisms

The following oral pathogens were studied: *Candida albicans* CBS 562 from “Centraalbureau voor Schimmelcultures” and bacteria *Streptococcus sanguis* ATCC 10556*, Streptococcus mitis* ATCC 903*, Porphyromonas gingivalis* ATCC 33277 and *Fusobacterium nucleatum* ATCC 25586 from American Type Culture Collection. The microorganisms were stored at −70°C in Sabouraud Dextrose Broth (SDB, Merck® - *C. albicans*) and Mueller-Hinton Broth (Difco® - bacteria) with 15% glycerol. It was considered the oxygen exigencies of each microorganism (*C. albicans* - aerobiosis, *S. mitis* and *S. sanguis* microaerophilie and *F. nucleatum* and *P. gingivalis* anaerobiosis) to choose bacteria growth conditions*.*

### Plant material

Twenty medicinal and aromatic species choose for this study were belonging to “Collection of Medicinal and Aromatic Plants” - CPMA of the Research Center for Chemistry, Biology and Agriculture (CPQBA), University of Campinas (UNICAMP), Campinas, SP, Brazil (http://www.cpqba.unicamp.br/), with the vouchers numbers indicated in Table 1. Samples were collected in spring/summer from November 2009 to January 2011, in the morning after dew point. The exsiccates from plant material used in this study are deposited in the herbarium of the Institute of Biology at UNICAMP - UEC and were identified by Dr. Washington Marcondes Ferreira Neto (curator). The species were deposited in the.

**Table 1:** **Medicinal and aromatic plants from CPMA – “Collection of Medicinal and Aromatic Plants” - CPQBA/UNICAMP selected for this study**

***Medicinal Species***	***Family***	***Popular Name***	***Source***	***No. CPMA***	No. Voucher*	***Popular use***
*Aloysia gratissima (*Giil & Hook)	Verbenaceae	Brazilian lavender	leaf	714	UEC 121.393	Digestive antispasmodic
*Aloysia triphylla* (L’Hér.) Britton	Verbenaceae	Aloisia	leaf	274/700	UEC 121.412	Sedative, antispasmodic
*Alpinia speciosa* (Pers.) Burtt & Smith	Zingiberaceae	Colony	root	447	UEC 145.185	Antimicrobial
*Baccharis dracunculifolia DC.*	Asteraceae	Broom weed	leaf	1841	-	Tonic, eupeptic, antipyretic
*Cinnamomun zeilanicus* Blume	Lauraceae	Cinnamon	leaf	455	IAC 19624	Carminative, antispasmodic
*Coriandrum sativum* L.	Apiaceae	Coriander	leaf	664	-	Antimicrobial,antifungal
*Cymbopogon citratus* (DC) Stapf	Poaceae	Lemon grass	leaf	503	UEC 85.210	Sedative, analgesic, anti-cough
*Cymbopogon martini* (Roxb.) J.F. Watson	Poaceae	Palmarosa	leaf	354	UEC 127.115	Antiseptic, antifungal
*Cymbopogon winterianus* Jowitt.	Poaceae	Lemon verbena	leaf	712	UEC 121.414	Repellent, insecticide
*Cyperus articulatus* Vahl	Cyperaceae	Piprioca	bulbs	222	UEC 121.396	Anti-inflammatory
*Elyonurus muticus* Spreng	Poaceae	Agripalma	leaf	1701	UEC 20.580	Antibacterial
*Eugenia florida* DC.	Myrtaceae	Guamirin-cereja	leaf	1685	IAC 49207	Anti-inflammatory
*Eugenia uniflora L*	Myrtaceae	Pitanga	leaf	1816	-	Anti-hypertensive, diuretic
*Lippia alba* (Mill) N.E. Brown	Verbenaceae	False lemon balm	leaf	467/509	UEC 121.413	Treatment of migraines
*Lippia sidoides* Cham.	Verbenaceae	Rosemary	leaf	398/399	-	Bactericide, fungicide
*Mentha* x *piperita* L.	Lamiaceae	Mint	leaf	560	UEC 127.110	Antifungal, antibacterial
*Mikania glomerata* Spreng	Asteraceae	Guaco	leaf	766	UEC 102.047	Anti-inflammatory, bronchodilator
*Siparuna guianenses* Aubl.	Monimiaceae	Wild lemon	leaf	2025	-	Tranquilizer, diuretic
*Syzygium aromaticum* (L.) Merr. & L. M. Perry	Myrtaceae	Cloves	leaf	455	IAC 19624	Seasoning, antibacterial
*Ziziphus joazeiro mart*	Rhamnaceae	Juazeiro fruit	leaf	2119	-	Astringent, Anti-inflammatory

*Deposited in the herbarium of species with no voucher number are being registered yet.

### EO extraction

The EOs were obtained from 100 g of fresh plant parts by water distillation for 3 h using a Clevenger-type system. After completion of this process, the system was cooled and the aqueous phase collected followed by washing of all the Clevenger apparatus with dichloromethane (50 ml) to obtain the EOs.The pooled organic phases were dried with sodium sulfate, filtered, and the solvent evaporated until dryness. The oil samples were stored at −25°C in sealed glass vials [16].

### Fractionation of EOs

For the fractionation, the oils were selected based on criteria such as good antimicrobial activity (MIC until 0.5 mg/mL [17], oil yield (*>*0.5%, except for *C. sativum* and *M. glomerata*), seasonal cultivation of the plants and potential production on commercial scale. The Eos (500 mg) were fractioned on a dry column (acetate cellulose 2 cm X 20 cm) prepared with silica gel 60 (20 gr / 0,063-0,200 mm) (Merck) by direct application onto the packed column, followed by elution. Dichloromethane was previously defined as mobile phase for the fractionation of the oils by TLC (Thin-Layer Chromatography) analysis of each EO using different eluents. After elution, the column was cut in different parts for each EO, and the fractions were individually transferred to appropriate flasks, dissolved in dichloromethane and re-analyzed by TLC. Fractions with similar chemical pattern were grouped resulting in fractions named F1, F2, F3 and F4 in order of increasing polarity. The resulting fractions were filtered under vacuum and the silica residue extracted with dichloromethane [16]. The fractions were analyzed using Gas chromatography coupled to mass spectrometry (GC-MS) and then used antimicrobial assays. All chemical wastes produced in the present study were treated according to the approval of Environmental Ethics Committee of UNICAMP (322/2009).

### Chemical Constituents of EOs and fractions - Gas chromatography–mass spectrometry (GC-MS)

The volatile constituents of the EOs oils were determined using a Hewlett-Packard 6890 gas chromatograph, equipped with a HP-5975 mass selective detector and HP-5MS capillary column (30 m × 0.25 mm × 0.25 μm diameter). GC and GC–MS were carried out using split (1:30) injection, with injector temperature set at 220°C, column at 60°C, a heating ramp of 3°C min^−1^ to a final temperature of 240°C, and the MS and FID detectors set at 250°C. Helium was used as the carrier gas at 1 mL min^−1^. The GC–MS electron ionization system was set at 70 eV. A sample of the EO was dissolved in ethyl acetate for the analyses. Retention indices (RI) relative to *n-*alkanes were calculated by linear interpolation. Oils components were identified by comparision of experimental RI with reference data [18], by maching mass spectra with NIST software 05® reference spectra and by injection of authentic standards, when available.

### Microbiological assays

#### Inocula preparation

*Candida albicans*, *S. sanguis*, *S. mitis*, *P. gingivalis* and *F. nucleatum* were grown overnight at 36°C in Sabouraud Dextrose Broth (SDB, Merck®) and Mueller-Hinton Broth (Difco®), respectively. The inocula were prepared according to CLSI protocols M27-A2 and M7-A6 (CLSI 2002, 2005) [19, 20]. The cells were diluted in 0.85% NaCl solution and the suspension turbidity adjusted to 0.5 on the McFarland scale and confirmed in a spectrophotometer (Shimadzu UV mini 1240 Spectrophometer) at 530 nm (*C. albicans*) or 625 nm (bacteria) to absorbance between 0.08-0.1 (10^6^ cells/mL for yeast and 10^8^ cells/mL for bacteria). The cell suspensions were finally diluted to 10^4^ cells/mL for (yeast) or 10^6^ cells/mL (bacteria).

#### Minimal Inhibitory Concentration (MIC) of the EOs

The MIC was determined using tissue culture microplates (96 wells) containing 100 μL of Brain Heart Infusion (BHI - Oxoib®), culture medium for bacteria and Sabouraud Dextrose Broth (SDB, Merck®) for *C. albicans*. The stock solutions of EOs oils and fractions were diluted to 4 mg/mL with propylene glycol, transferred to the first well and serial dilutions were performed to reach concentrations ranging from 1.0-0.00048 mg/mL. Nystatin (Sigma® - 1%) and chlorhexidine digluconate (Sigma® - 0.12%) were used as antimicrobial standard. The inocula (100 μL) was added to all the wells, and the plates incubated at 36°C for 48 h in aerobic, microaerophilic or anaerobic conditions. The MIC was defined as the lowest concentration of the EO able to prevent the microbial growth. The tests were performed in three independent experiments, each one in triplicate [19, 20].

#### Minimal Bactericidal/Fungicidal Concentration (MBC/MFC) of the EOs

Based on the MIC results 10 μL of the cells suspension from the wells showing no visible microbial growth and from three wells above them were subcultured in Petri dishes containing Sabouraud Dextrose Agar medium (SDA- Merck®) for yeasts and Tryptic Soy Agar (TSA-Difco®) or Blood Agar media for bacteria. The plates were incubated at 36°C until five days in aerobic, microaerophilic or anaerobic conditions. The MBC/MFC was defined as the lowest sample concentration showing no cell growth on the inoculated agar surface. The tests were performed in three independent experiments, each one in triplicate [19, 20]. The EOs and fractions antimicrobial activity was classified in strong, moderate or weak according to Duarte et al. [17].

### Biofilms assays

#### Mode of action of the EOs on biofilm

The method employed for biofilm studies was carried out according to described in the antimicrobial assays (MIC) with modifications. The inocula from cultures were prepared at 10^7^ cells/mL for bacteria and 10^5^ cells/mL for *C. albicans* in BHI or SDB media, respectively, enriched with 2% sucrose. The cultures were incubated at 36°C for 72 h under appropriate atmosphere in order to promote microbial adherence to the bottom of the wells. Subsequently, MIC values were confirmed and to determine MBC/MFC, each adhered biofilm was transferred by swab technique to the surface of blood agar or SDA agar in petri dishes and incubated at 30°C for until five days according to oxygen microorganisms requirements [19, 20]. The MIC/MBC/MFC values were used to determine the MBC:MIC or MFC:MIC ratio, as previously proposed by Hafidh *et al*. [21] to establish the nature of antimicrobial effect, regard to inhibition or killing of the tested microorganisms.

#### Effect of the EOs and fractions on biofilm formation

The biofilms were carried out using sterile untreated 96-well polyethylene U-bottom plates (IPT) containing the specific medium (Sabouraud for yeast and BHI for bacteria) enriched with 2% sucrose. The EOs and fractions were diluted with propylene glycol (4 mg/mL), transferred to the first well and serial dilutions were performed to reach concentrations ranging from 1.0-0.0048 mg/mL. The 1% Nystatin (Sigma®) and 0.12% chlorhexidine digluconate (Sigma®) solutions were used as antibiotic standard. After this procedure, microbial cells (1.0 × 10^5^ cells/mL for yeasts and 1.0 × 10^7^ cells/mL for bacteria) were added to the wells and the plates were incubated at 36°C for 72 h [22].

#### Biofilm quantification

Following biofilm formation the medium was aspirated and no adhered cells were removed by washing the wells twice with 200 μL of distilled water. The plates were then dried at room temperature for 45 min. One hundred microliters of a 0.4% crystal violet solution was added to all the wells. After 45 min, the biofilms formed in the bottom of the wells were washed four times with distilled water and immediately distained with 200 μL of 95% ethanol. After a further 45 min, 100 μL of well solution were transferred to a well in a new plate and the absorbance measured at 595 nm in a microplates reader (Asys - Expert Plus). The amount of biofilm formed was calculated by subtracting the absorbance values from control well [23].

### Scanning Electron Microscopy (SEM) of biofilms

In order to assess the integrity of the microbial cells, biofilms were developed in a Lab-Tek TM coverslip chambers (Nunc) as described above, and treated with standard drugs and EOs at 1 mg/mL. The samples were washed twice with 3% glutaraldehyde in phosphate buffer (pH 7.4) and fixed in glutaraldehyde 0.15 M 2.5% (v/v) at room temperature for 12 h. The dehydrated cells were submitted to sequential baths of ethanol at concentrations of 50%, 70%, 90% and absolute ethanol twice, until the dried at the critical point, then coated with gold in a Metalizer and observed using a Scanning Electron Microscope (Jeol model JSM 5600 Lv) [24].

### Statistical analysis

Statistical analysis was performed with one-way ANOVA and p-values ≤0.05 considered statistically significant. The inhibition of biofilm formation data were compared by Tukey test. The statistical test was run using STATISTICA® v.8.0 (Stafsoft, USA) system software.

## Results and discussion

### Oil and fractions yields

The EOs and fractions yields are presented in Tables 2 and 3, respectively, relative to mass of dry plant material. The highest oil yields were obtained from *L. sidoides* (4.67%), *M. piperita* (2.22%), *C. winterianus* (1.48%), *C. citratus* (1.13%) and *A. gratissima* (1.10%).

**Table 2:** **Oil yield and antimicrobial activity of the EOs studied against oral pathogens (MIC/MBC/MFC – mg/mL)**

		***Microorganisms***									
***Medicinal species***	***% Yield*** (%)	***C. albicans*** CBS 562		***F. nucleatum*** ATCC 25586		***P. gingivalis*** ATCC 33277		***S. sanguis*** ATCC 10556		***S. mitis*** ATCC 903	
		MIC	MFC	MIC	MBC	MIC	MBC	MIC	MBC	MIC	MBC
***Aloysia gratissima (Aff & Hook).Tr***	1.10	0.015	0.062	0.125	0.250	0.125	0.125	0.500	1.000	0.250	0.250
***Aloysia triphylla (L’Hér.) Britton***	0.27	0.015	0.062	0.125	0.250	0.250	0.250	0.500	1.000	0.500	0.500
***Alpinia speciosa (Pers.) Burtt & Smith***	0.22	*0.007*	*0.062*	0.125	0.125	0.125	0.250	0.500	*	0.500	*
***Baccharis dracunculifolia DC.***	0.80	0.250	0.500	0.125	0.250	0.125	0.125	0.500	0.500	0.250	0.250
***Cinnamomun zeilanicus Blume***	0.22	*0.007*	*0.007*	0.250	0.250	0.250	0.250	0.500	1.000	0.500	0.500
***Coriandrum sativum L.***	0.29	*0.007*	*0.015*	*0.015*	*0.125*	0.125	0.125	0.250	0.500	*0.062*	*0.125*
***Cymbopogon citratus (DC) Stapf***	1.13	0.015	0.125	0.250	0.250	0.250	0.250	0.500	*	0.250	0.500
***Cymbopogon. martini (Roxb.) J.F. Watson***	0.59	0.015	0.125	0.125	0.250	0.250	0.250	0.500	*	0.250	0.250
***Cymbopogon. winterianus Jowitt.***	1.48	0.015	0.125	0.125	0.250	0.250	0.500	0.500	*	0.250	0.500
***Cyperus articulatus L.***	0.50	0.125	0.500	0.250	0.250	0.250	0.250	0.250	0.500	0.250	0.500
***Elyonurus muticus Spreng.***	0.61	0.250	*	0.250	0.500	0.250	0.250	0.500	1.000	0.500	*
***Eugenia florida DC.***	0.34	0.125	*	0.125	0.250	0.125	*	0.125	0.250	0.500	0.500
***Eugenia uniflora L***	0.76	0.250	*	0.125	0.125	0.250	0.250	0.500	0.500	0.500	0.500
***Lippia alba (Mill) N.E. Brown***	0.30	0.250	0.500	0.125	0.125	0.250	0.250	0.250	1.000	0.250	*
***L. sidoides Cham.***	4.67	0.250	0.500	0.125	0.125	0.250	0.250	0.125	0.500	0.250	*
***Mentha piperita L.***	2.22	0.500	*	0.250	0.250	0.250	*	0.500	0.500	0.500	0.500
***Mikania glomerata Spreng***	0.40	0.250	0.250	0.250	0.500	0.500	*	*0.062*	*0.125*	0.125	0.125
***Siparuna guianenses Aubl***	0.29	0.125	0.250	0.062	0.250	*0.062*	*0.125*	0.250	1.000	0.125	0.250
***Syzygium aromaticum (L.) Merr. & L. M. Perry***	0.46	0.500	0.500	0.250	0.250	0.250	0.250	0.500	1.000	0.500	0.500
***Ziziphus joazeiro mart***	0.46	1.000	*	0.250	0.500	0.250	0.250	0.500	1.000	0.500	0.500
**Nystatin**	-	0.007	0.015	-	-	-	-	-	-	-	-
**Chlorhexidine digluconate**		-	-	0.015	0.015	0.015	0.125	0.015	0.015	0.015	0.125

*Fungicidal/bactericidal action: MIC > 1 mg/mL.

**Table 3:** **Antimicrobial activity of the crude EO and their fractions against oral pathogens (MIC/MBC/MFC – mg/mL)**

		***Microorganisms***									
***Medicinal species***	***% Yield fraction***	***C. albicans*** CBS 562		***F. nucleatum*** ATCC 25586		***P. gingivalis*** ATCC 33277		***S. sanguis*** ATCC 10556		***S. mitis*** ATCC 903	
		MIC	MFC	MIC	MBC	MIC	MBC	MIC	MBC	MIC	MBC
***EO A. gratissima***		*0.015*	*0.062*	0.125	0.250	*0.125*	*0.125*	0.500	1.000	0.250	0.250
***F***_***1***_ ***AG***	25.70	0.500	1.000	0.500	0.500	0.250	0.500	0.500	1.000	0.250	0.500
***F***_***2***_ ***AG***	11.12	0.500	*	0.500	0.500	0.250	0.500	0.500	1.000	0.500	0.500
***F***_***3***_ ***AG***	26.94	0.500	1.000	0.250	0.500	0.250	0.500	0.500	0.500	0.250	0.500
***F***_***4***_ ***AG***	16.48	0.125	*	*0.062*	*0.250*	0.125	0.500	*0.125*	*0.125*	*0.125*	*0.125*
***EO C. sativum***		*0.007*	*0.015*	*0.015*	*0.125*	*0.125*	*0.125*	*0.250*	*0.500*	*0.063*	*0.125*
***F***_***1***_ ***CS***	24.88	0.500	1.000	0.250	0.250	0.125	0.500	0.500	0.500	0.500	0.500
***F***_***2***_ ***CS***	39.20	0.250	1.000	0.125	0.250	0.125	0.500	0.500	0.500	0.250	1.000
***F***_***3***_ ***CS***	15.20	O.250	1.000	0.250	0.250	0.125	0.500	0.500	*	0.250	1.000
***F***_***4***_ ***CS***	9.20	0.250	1.000	*	*	0.500	1.000	0.500	1.000	0.500	*
***EO C. articulatus***		*0.125*	*0.500*	0.250	0.250	0.250	0.250	*0.250*	*0.500*	0.250	0.500
***F***_***1***_ ***CA***	9.20	0.250	1.000	0.250	*	*0.125*	*0.250*	*	*	0.500	*
***F***_***2***_ ***CA***	9.41	0.250	*	0.250	0.250	0.500	1.000	1.000	*	0.500	*
***F***_***3***_ ***CA***	24.51	0.250	*	0.250	0.250	0.250	1.000	0.500	1.000	0.250	0.500
***F***_***4***_ ***CA***	26.16	0.250	1.000	*0.125*	*0.250*	0.250	0.250	0.250	0.500	*0.250*	*0.250*
***EO M. glomerata***		*0.250*	*0.250*	0.250	0.500	0.500	*	*0.062*	*0.125*	0.125	0.125
***F***_***1***_ ***MG***	42.80	0.250	*	0.250	0.500	0.250	0.500	*	*	0.500	*
***F***_***2***_ ***MG***	14.00	0.250	0.500	0.250	*	0.500	0.500	*	*	0.500	1.000
***F***_***3***_ ***MG***	7.20	0.250	1.000	0.250	*	0.250	0.500	*	*	*	*
***F***_***4***_ ***MG***	2.56	0.250	1.000	0.250	*	*0.250*	*0.250*	0.250	1.000	0.250	0.500
***EO L. sidoides***		*0.250*	*0.500*	0.125	0.125	*0.250*	*0.250*	0.125	0.500	0.250	*
***F***_***1***_ ***LS***	31.96	0.250	*	0.062	0.250	0.250	*	0.500	0.500	0.250	*
***F***_***2***_ ***LS***	43.12	0.500	1.000	*0.031*	*0.250*	0.125	*	*0.125*	*0.250*	*0.125*	*0.125*
***F***_***3***_ ***LS***	13.34	0.500	0.500	0.062	0.250	0.250	0.500	0.250	0.250	0.125	0.250
***F***_***4***_ ***LS***	4.96	0.250	1.000	0.125	0.250	0.250	*	0.250	0.500	0.250	*
**Nystatin/**		0.007	0,015	-	-	-	-	-	-	-	-
**chlorhexidine digluconate**		-	-	0.015	0.015	0.015	0.125	0.015	0.015	0.015	0.125

*Fungicidal/bactericidal action: MIC > 1 mg/mL ,

AG: *A. gratissima,* CA: *C. articulatus*, CS: *C. sativum,* MG: *M. glomerata,* LS: *L. sidoides.*

### Antimicrobial assays

The results obtained for MIC/MBC/MFC of the EOs against the oral microorganisms are shown in Table 2. According to Duarte et al. [17], the EOs presented strong to moderate antimicrobial activity against planktonic cells, with MIC values between 0.007 and 1.00 mg/mL. The highest activities were observed for *A. speciosa, C. sativum* and *C. zeilanicus* EOs against *C. albicans* (0.07 mg/mL)*. Coriandrum sativum* oil inhibited *F. nucleatum* and *S. mitis* at lowest MIC values (0.015 mg/mL and 0.062 mg/mL, respectively) compared with the other oils tested. *S. guianenses* and *M. glomerata* oils inhibited, respectively, the growth of *P. gingivalis* and *S. sanguis* at 0.062 mg/mL.

According to the criteria previously mentioned, the oils from *A. gratissima* (AG)*, C. articulatus* (CA), *C. sativum* (CS), *L. sidoides* (LS) and *M. glomerata* (MG) were fractionated and the fractions were submitted to antimicrobial assays. The MIC/MBC/MFC results from crude oils (EO) and fractions are shown in Table 3. The highest inhibitory and bactericidal effects presented by the fractions were observed for F2LS (Fraction 2 from *L. sidoides*) against *F. nucleatum* and *S. mitis* and for F1CA (fraction 1 from *C. articulatus*) and F4AG (fraction 4 from *A. gratissima*) against *P. gingivalis* and *S. sanguis,* respectively. However, all the fractions presented similar or lower activity than the crude oil against the microorganisms suggesting a synergistic action from the compounds present in crude oil.

The marked activity found for some oils in the present study was previously verified for bacteria such as *Bacillus megaterium*, *B. cereus*, *S. piogenes*, *Escherichia coli* and *Proteus mirabilis* (*C. articulatus*) and standards or clinical isolates of *Candida* spp (*C. sativum*) [25, 26]. The oil from *L. sidoides* obtained in this study inhibited *S. mitis* and *S. sanguis* at MIC values higher than those observed by Botelho *et al*. [27] in an analogous study.

### Mode of action of the EOs and fractions on biofilm

The results of MIC/MBC/MFC for the biofilms as well as the MBC/MFC:MIC ratio of the most active crude oils and fractions are shown in Table 4. The ratio calculation was adapted from Hafidh *et al*. [21]. According to the results, *C. articulatus* EO stood out, inhibiting all investigated species with the lowest MIC/MBC values. Similar activities were observed for the crude oil and the F2LS from *L. sidoides* against *S. sanguis*, and for *A. gratissima* oil against *S. mitis* biofilm*.* As expected, planktonic cells from all strains studied were more susceptible to the EOs (Table 2) when compared to biofilm (Table 4) as observed in previous studies to a great variety of antimicrobial agents [28, 29]. Chandra [30] observed that antifungal agents used against *C. albicans* biofilms were much less active than against planktonic cells, and that the concentrations required to reach 50% inhibition of the metabolic activity were around 5 to 8 times higher. Also, *Eucalyptus* oil and its major component 1,8-cineole, when employed alone or combined with chlorhexidine digluconate against biofilms from several microorganisms cultures including *C. albicans,* showed better activities against planktonic cells [31]. The results confirm the effective action of *C. articulatus* EO that exerted bactericide/fungicide action against all oral microorganisms studied. The mode of action observed for *C. articulatus* EO on the microorganisms studied can be related to the possible mechanisms of action presented by its major components, a- and b-pinene (Table 5) that showed be able to destroy cell integrity, and inhibit respiration and the ion transport processes, leading to cell death [32]. Besides, the *C. articulatus* compounds presented a considerable antibacterial effect, especially on a methicilline-resistant *Staphylococcus aureus* and on Gram-positive and Gram-negative bacteria [33].

**Table 4:** **Antimicrobial activity of the EO against biofilms (72 h) and MBC/MFC:MIC ratio**

	Microorganisms														
***Medicinal species***	***C. albicans*** CBS 562			***F. nucleatum*** ATCC 25586			***P. gingivalis*** ATCC 33277			***S. sanguis*** ATCC 10556			***S. mitis*** ATCC 903		
	***MIC***	***MFC***	***Ratio***	***MIC***	***MBC***	***Ratio***	***MIC***	***MBC***	***Ratio***	***MIC***	***MBC***	***Ratio***	***MIC***	***MBC***	***Ratio***
***A. gratissima***	0.500	*	ND	0.500	0.500	1:1	0.125	*	ND	0.500	1.000	2:1	*0.250*	*0.500*	*2:1*
***F***_***4***_ ***AG***	0.500	*	ND	0.250	1.000	4:1	0.500	*	ND	0.500	0.500	1:1	0.250	*	ND
***C. sativum***	0.250	1.000	4:1	0.250	*	ND	0.500	*	ND	0.500	*	ND	0.500	*	ND
***M. glomerata***	0.500	0.500	1:1	0.250	*	ND	1.000	*	ND	0.500	*	ND	0.500	*	ND
***F***_***4***_ ***MG***	1.000	1.000	1:1	0.500	1.000	2:1	*	*	ND	0.500	0.500	1:1	0.500	*	ND
***C. articulatus***	*0.250*	*0.250*	*1:1*	*0.250*	*0.500*	*2:1*	*0.250*	*0.500*	*2:1*	*0.250*	*0.500*	*2:1*	*0.500*	*1.000*	*2:1*
***F***_***4***_ ***CA***	1.000	*	ND	0.250	*	ND	*	*	ND	0.500	0.500	1:1	0.500	*	ND
***L. sidoides***	0.500	1.000	2:1	0.500	1.000	2:1	0.500	*	ND	0.250	0.500	2:1	0.500	*	ND
***F***_***2***_ ***LS***	0.500	*	ND	0.250	1.000	4:1	0.250	*	ND	0.250	0.500	2:1	0.500	*	ND
**Nystatin**	1.000	*	ND	-	-	-	-	-	-			-	-	-	-
**Chlorhexidine**	-	-	-	0.015	0.125	8:1	0.015	0.125	8:1	0.015	0.015	1:1	0.015	0.062	4:1

*Fungicidal/bactericidal action: MIC > 1 mg/mL.

ND- Not determined.

Ratio: MBC:MIC or MFC:MIC between 1:1–2:1 - bactericidal or fungicidal effect, while ratio >2:1 -a bacteriostatic or fungistatic effect.

**Table 5:** **Compounds identified in the active EO and fractions**

Compounds ^a^	RI ^b^	AG ^c^	CA	CS	MG	LS	F _4_ AG	F _4_ CA	F _4_ MG	F _2_ LS
*Z*-3-hexen-1-ol	857			5.11						
*E*-2-hexen-1-ol	868			2.17						
Cyclohexanone	899					6.50				
Nonane	901			2,70						
a-pinene	933		5.72							
b-pinene	977	12.01	3.52							
p-cymene	1024		0.73			17.28				
Limonene	1027	1.51	1.12		2.06					
Linalool	1101	0.49		0.77			2.62			
*E*-pinocarveol	1138	2.96	4.44				13.16	21.97		
*E*-verbenol	1144	1.59	2.38					8.43		
*E*-sabinol	1145						4.29			
*E*-pinocamphone	1161	16.07					0.84			
*a*-phellandrene-8-ol	1167		1.75							
p-menta-1,5-dien-8-ol	1169		0.67					8.97		
*Z*-pinocanphone	1173	6.04					0.46			
p-cymen-8-ol	1186		0.51				0.93	2.97		
a-terpineol	1192		0.30				0.95	2.17		
Myrtenal	1195		2.37					9.18		
Myrtenol	1196	1.81	2.13				5.31			
Verbenone	1208		1.67					9.73		
*E*-carveol	1220						1.06	2.29		
Geraniol	1264									
1-decanol	1269			33,91						
*E*-2-decen-1-ol	1271			23,59						
Geranial	1272									
Thymol	1290					65.76		0.60		97.20
*E*-pinocarveol acetate	1299	8.19								
a-copaene	1373		4.97		0.76					
a-caryophyllene	1416	7.19			9.49	10.46				
a-guaiene	1436		2.17							
2-dodecen-1-ol	1469			13,06						
g-muurolene	1477	3.79							3.39	
Germacrene D	1481				38.29					
b-selinene	1482		2.96							
a-selinene	1491		2.46							
Bicyclogermacrene	1493	4.20			7.98					
a-bulnesene	1503		5.02							
Elemol	1547	0.48			0.94		1.64		7.39	
Germacrene B	1552				3.35					
Sphatulenol	1574	1.54			3.65		3.96		4.31	
Caryophyllene oxide	1578	2.60	3.41		4.28		1.85	0.55	5.63	0.50
Guaiol	1596	8.53					29.63			
Isolongifolan-7-a-ol	1620				1.21				11.58	
Muurola-4,10-dien-1-b-ol	1628				3.59				10.42	
a-muurolol	1646						0.77		3.45	
b-cadinol	1652				3.47				25.85	
Bulnesol	1665	3.14					11.79			
Tetradecanol	1670			2,92						
*E*-2-tetradecen-1-ol	1674			5,46						
Mustakone	1675		5.66							
Ishwarone	1680		1.51					8.80		
Germacra-4(15),5,10(14)-trien-1-a-ol	1684								5.13	
Eudesma-4(15),7-dien-1-b-ol	1685								9.85	
Others compounds identified <2%	-	10.21	7.58	8.04	8.32	-	6.55	7.39	3.77	2.31
Total		92.35	63.05	97.73	87.39	100	86.04	85.18	90.56	99.80

^a^Mw = molecular weight;

^b^RI = retention índex;

^c^Results expressed as % of area. AG: *A. gratissima,* CA: *C. articulatus*, CS: *C. sativum,* MG: *M. glomerata,*

LS: *L. sidoides,* F_4_MG: F_4_ *M. glomerata*, F_4_CA: F_4_ *C. articulatus,* F_4_AG: F_4_ *A. gratissima,* F_2_LS: F_2_ *L. sidoides.*

Regard to the inhibition of biofilm formation in the presence of 1 mg/mL of EOs and fractions (Table 6), the fraction F2LS and the oils from *A. gratissima* and *C. articulatus* demonstred the highest inhibition, respectively, on *F. nucleatum* (62.29%) and *P. gingivalis* (44.41%), *S. mitis* (9%) and *C. albicans* (28.08%) and *S. sanguis* (63.96%). Since no significative difference (p ≤ 0,05) was observed between the action of the *C. articulatus* crude oil and the fraction F2LS against *F. nucleatum* and *P. gingivalis* biofilms, the crude oils from *C. articulatus* and *A. gratissima* was chosen for further assays in the range of 0.0048 – 1 mg/mL (Table 7). In this condition, *A. gratissima* inhibited the formation of *S. mitis* biofilm only at 1 mg/mL (9%), even though this activity was superior to that of the chlorhexidine. On the other hand, the biofilm inhibition by *C. articulatus* oil was proportional to the concentration employed, and also similar or superior to standards used.

**Table 6:** **Inhibition of biofilm formation (%) of the oral microorganisms in the presence of the EOs and fractions at 1 mg/mL**

***Microorganisms***					
***Medicinal species***	***C .albicans*** CBS 562	***F. nucleatum*** ATCC 25586	***P. gingivalis*** ATCC 33277	***S. sanguis*** ATCC 10556	***S. mitis*** ATCC 903
***EO A .gratissima***	12.31 _f_	55.83 _d_	39.12 _a_	60.83 _a,b_	**9.00**_a_
***F***_***4***_ ***AG***	19.23 _d,e_	56.46 _d_	30.88 _b_	58.13_b,c_	8.50 _a_
***EO C. sativum***	23.08 _c,d_	55.83 _d_	39.71 _a_	58.33 _a,b,c_	1.50 _b_
***EO M. glomerata***	22.69 _c,d_	58.96 _c_	40.00 _a_	54.79 _c_	1.00 _b_
***F***_***4***_ ***MG***	20.77_d,e_	60.83 _b_	37.94 _a_	60.63 _a,b,c_	0.00 _c_
***EO C. articulatus***	*28.08*_*a,b*_	*61.67*_*a,b*_	*43.53*_*a*_	*63.96*_*a*_	5.00 _a,b_
***F***_***4***_ ***CA***	25.77 _a,b,c_	61.25 _a,b_	39.41 _a_	61.67 _a,b_	2.50 _b_
***EO L. sidoides***	16.55 _e,f_	58.33 _c_	12.94 _c_	58.13 _a,b,c_	5.50 _a,b_
***F***_***2***_ ***LS***	23.85 _b,c,d_	*62,29*_*a*_	*44.41*_*a*_	42.71 _d_	0.00 _c_
**Nystatin/**	29.62 _a_	_	_	_	_
**Chlorhexidine digluconate**	_	55.42 _d_	37.65 _a_	57.08 _c_	1.50 _b_

AG: *A. gratissima,* CA: *C. articulatus*, CS: *C. sativum,* MG: *M. glomerata,* LS: *L. sidoides.* Values of 0.00 indicates any inhibition on biofilm formation.

Values in the same column with different letters (a-f) are significantly different (p≤0,05) by Tukey Test.

**Table 7:** **Biofilm Inhibition (%) of the oral microorganisms in the presence of** ***C. articulatus*** **and** ***A. gratissima*** **EO**

	***Microorganism***									
***Concentration***	***C .albicans*** CBS 562		***F. nucleatum*** ATCC 25586		***P. gingivalis*** ATCC 33277		***S. sanguis*** ATCC 10556		***S. mitis*** ATCC 903	
	***C. articulatus***	***Nystatin***	***C. articulatus***	***Chlorhexidine***	***C. articulatus***	***Chlorhexidine***	***C. articulatus***	***Chlorhexidine***	***A. gratissima***	***Chlorhexidine***
1	28.08	29.62	61.67	55.42	43.53	37.65	63.96	57.08	9.00	1.50
0.500	14.23	28.72	54.79	56.46	32.35	37.94	58.96	46.46	0.00	3.50
0.250	22.69	20.77	60.42	55.21	28.24	35.59	54.17	55.83	0.00	0.00
0.125	19.23	27.69	57.50	55.42	0.00	39.12	23.13	57.92	0.00	0.00
0.062	21.54	23.08	49.17	53.54	0.00	37.06	7.29	56.25	0.00	0.00
0.031	19.23	21.15	44.38	55.83	0.00	29.41	10.02	56.04	0.00	0.00
0.015	19.62	13.08	40.21	55.21	0.00	13.82	10.63	53.54	0.00	0.00
0.007	16.92	16.15	31.67	42.50	0.00	1.76	4.38	51.46	0.00	0.00
0.003	14.62	0.00	0.00	52.92	0.00	0.00	0.00	47.08	0.00	0.00
0.0019	17.69	0.00	0.00	48.33	0.00	0.00	0.21	50.63	0.00	0.00
0.00097	4.62	0.00	0.00	53.96	0.00	0.00	0.00	27.08	0.00	0.00
0.00048	0.00	0.00	0.00	54.17	0.00	0.00	3.54	18.33	0.00	0.00

The values indicated in the table 0.00 demonstrated that don’t have any inhibition on biofilm formation.

### Scanning electronic microscopy (SEM)

**Figure 1:**
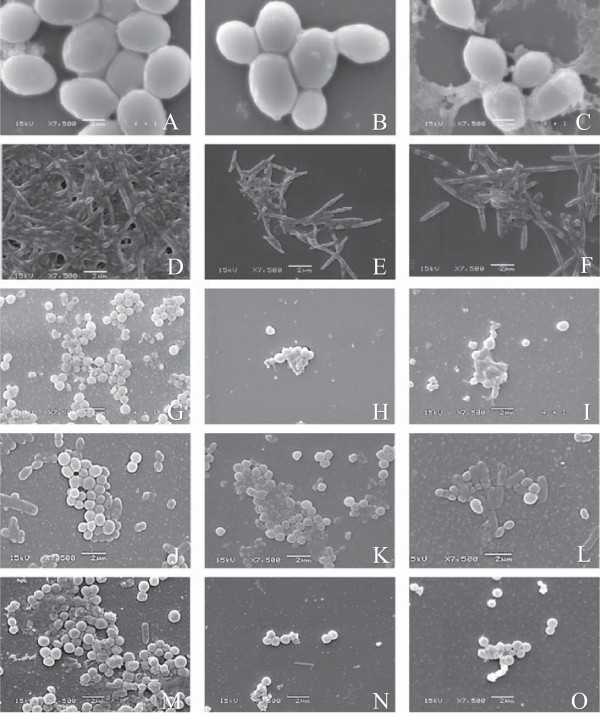
**Scanning electron micrographs of biofilms - 7.500x. (A)** *C. albicans*; **(B)** *C. albicans* in the presence of *C. articulatus* oil; **(C)** *C. albicans* in the presence of Nystatin; **(D)** *F. nucleatum*; **(E)** *F. nucleatum* in the presence of *C. articulatus* oil; **(F)** *F. nucleatum* in the presence of chlorhexidine digluconate. **(G)** *P. gingivalis*; **(H)** *P. gingivalis* in the presence of *C. articulatus* oil; **(I)** *P. gingivalis* in the presence of chlorhexidine digluconate; **(J)** *S. mitis*; **(K)** *S. mitis* in the presence of *A. gratissima* oil; **(L)** *S. mitis* in the presence of chlorhexidine digluconate; **(M)** *S. sanguis*; **(N)** *S. sanguis* in the presence of *C. articulatus* oil; **(O)** *S. sanguis* in the presence of chlorhexidine digluconato.

Morphological alterations in the microorganisms cells and biofilm arrangement exposed to the EOs were investigated by SEM (Figure 1). Through the SEM images was possible to observe reduction of biofilm formation and changes in the conformational structure probably due to a decrease in the cells adherence and consequently in the biofilm formation. These changes were also observed by Galvão *et al*. [34] whose tested the action of the EOs and bioactive fractions against *S. mutans*. However, apparently the EOs do not appear to have caused changes at cellular level. The decrease in the ability to form biofilm can be explained by the occurrence of various resistance mechanisms, which are still not completely understood given by the expression of resistance genes, and which can be attributed to a decrease in the rate of cell growth, particularly to those situated close to the adherence surface [35].

### Chemical composition of the active EOs and fractions

The major compounds identified in the most active oils and fractions are shown in Table 5. The analysis showed the presence of derived from aliphatic alchools in the *C. sativum* oil such as 1-decanol, *E*-2-decen-1-ol, 2 dodecen-1-ol, *E*-2-tetradecen-1-ol, *E*-3-hexen-1-ol, previously described in this aromatic specie commonly used in Brazilian culinary [36]. Anti-*Candida* activity of these compounds was comproved by Furletti *et al.*[25], which tested standards and correlated the activity to *Z*-2-hexen-ol, *E*-2-hexen-ol, *E*-3-hexen-ol and 1-decanol.

The main compounds identified in the other active oils were b-pinene, E-pinocamphone, *E*-caryophyllene, *E*-pinocarveol acetate and guaiol in *A. gratissima* and, a-pinene, a-bulnesene, *E*-pinocarveol and a-copaene in *C. articulatus*. Regarding to these compounds several authors have already shown their efficacy against both Gram-positive and Gram-negative bacteria [37–40].

The action mechanisms of the EOs and its compounds are not yet fully elucidated, but includes the inhibition of proton motive force and electron transfer and, consequently inhibition of the respiratory chain, mechanism of transport and decrease in substrate oxidation and membrane damage, leading to cell death [41–44]. Further studies should be developed in order to investigate the mechanisms by which the oils and their compounds acted on the oral microorganisms in the present study.

## Conclusion

The action of *C. sativum* EO against planktonic cells of *C. albicans* stood out from others EOs showing the lowest MIC values against the oral microorganisms investigated. The crude *C. articulatus* oil showed the highest inhibition on the cells adherence and consequently in the biofilms formation. The oils from these plants can be considered as new sources of antibacterial agents with great potential against oral pathogens.
